# Liquid flow in scaffold derived from natural source: experimental observations and biological outcome

**DOI:** 10.1093/rb/rbac034

**Published:** 2022-05-30

**Authors:** Elisabetta Salerno, Giulia Orlandi, Claudio Ongaro, Alessandro d’Adamo, Andrea Ruffini, Gianluca Carnevale, Barbara Zardin, Jessika Bertacchini, Diego Angeli

**Affiliations:** CNR-NANO S3 Research Center on Nanostructures and Biosystems at Surfaces, Modena I-41125, Italy; Department of Sciences and Methods for Engineering, University of Modena and Reggio Emilia, Reggio Emilia 42122, Italy; Department of Surgery, Medicine, Dentistry and Morphological Sciences with Interest in Transplant, Oncology and Regenerative Medicine, University of Modena and Reggio Emilia, Modena 41125, Italy; DIEF-Engineering Department “Enzo Ferrari”, University of Modena and Reggio Emilia, Modena 41125, Italy; DIEF-Engineering Department “Enzo Ferrari”, University of Modena and Reggio Emilia, Modena 41125, Italy; Institute of Science and Technology for Ceramics (ISTEC), National Research Council (CNR), Faenza 48018, Italy; Department of Surgery, Medicine, Dentistry and Morphological Sciences with Interest in Transplant, Oncology and Regenerative Medicine, University of Modena and Reggio Emilia, Modena 41125, Italy; DIEF-Engineering Department “Enzo Ferrari”, University of Modena and Reggio Emilia, Modena 41125, Italy; Department of Surgery, Medicine, Dentistry and Morphological Sciences with Interest in Transplant, Oncology and Regenerative Medicine, University of Modena and Reggio Emilia, Modena 41125, Italy; Department of Sciences and Methods for Engineering, University of Modena and Reggio Emilia, Reggio Emilia 42122, Italy

**Keywords:** fluid shear stress, flow resistance, hydroxyapatite, 3D scaffold

## Abstract

This study investigates the biological effects on a 3D scaffold based on hydroxyapatite cultured with MC3T3 osteoblasts in response to flow-induced shear stress (FSS). The scaffold adopted here (B-HA) derives from the biomorphic transformation of natural wood and its peculiar channel geometry mimics the porous structure of the bone. From the point of view of fluid dynamics, B-HA can be considered a network of micro-channels, intrinsically offering the advantages of a microfluidic system. This work, for the first time, offers a description of the fluid dynamic properties of the B-HA scaffold, which are strongly connected to its morphology. These features are necessary to determine the FSS ranges to be applied during *in vitro* studies to get physiologically relevant conditions. The selected ranges of FSS promoted the elongation of the attached cells along the flow direction and early osteogenic cell differentiation. These data confirmed the ability of B-HA to promote the differentiation process along osteogenic lineage. Hence, such a bioactive and naturally derived scaffold can be considered as a promising tool for bone regeneration applications.

## Graphical Abstract

**Figure:**
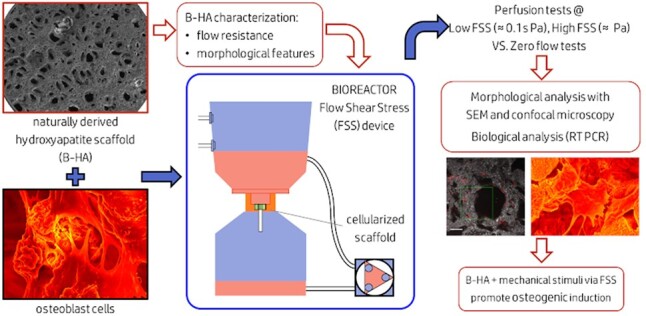

## Introduction

Bone is a mineralized and viscous-elastic connective tissue, which exerts important functions in the body, such as support, protection of other tissues and mineral storage [1]. This tissue is constantly remodeled in physiological conditions and it has an effective regenerative ability for the self-repair of bone defects, thanks to the coordinated actions of osteoclasts, osteoblasts, osteocytes and bone lining cells. However, in case of critical-sized and non-union defects in long segments of load-bearing bones, the repair process can be compromised such that the damaged area cannot fully and spontaneously regenerate [2].

The medical treatment of bone fractures and defects currently relies on a large variety of solutions and implant materials [3]. Among them, scaffold-based tissue engineering represents a promising solution for bone regenerative and surgical purposes [4, 5]. Many synthetic materials have been created up to now that can offer multiple choices in terms of adaptability to specific chemical, physical and mechanical requirements. However, one of their main limitations is that they cannot mimic the highly organized structure of the natural bone in macro- and micro-channels to guide cell migration, proliferation and differentiation in new forming bone tissue [6, 7].

On the opposite side, scaffolds obtained by natural sources possess unique structural characteristics and a high resemblance to autologous bone, which make them optimal solutions for implantation [8]. 3D scaffolds based on hydroxyapatite achieved from the biomorphic transformation of the natural wood are part of this last category. Such a material has been recently developed by means of a multi-step process, which allowed a total conversion of organic material to hydroxyapatite phase, while maintaining the original native macro-, micro- and nanostructure [9]. The so derived hydroxyapatite scaffold will be hereinafter named as B-HA, where ‘B’ represents three characteristic groups of peaks typical of the carbonation in B position of hydroxyapatite. The hierarchical structure, the distribution of the pores, their interconnection and the presence of apatite nanocrystals play a central role in regulating cell adhesion, habitability, and spatial distribution, thus influencing the cellular signal expression and, consequently, tissue formation. Excellent mechanical, structural and osteoconductive properties were found for B-HA [8–11].

Another important aspect to be considered when evaluating the suitability of a bioactive scaffold is the impact of fluid shear stress (FSS) on the seeded cells. In fact, the transformation of mechanical stimuli into biochemical responses of the cells is at the basis of both bone formation, repair and regeneration, and adaptation of the skeleton to its external environment [12–14]. In bones, fluid shear stresses are generated over the cells by the interstitial fluid flow, which modifies according to bone deformations, as a consequence of mechanical stimulation. In turn, through the pericellular matrix, FSS induces strains in the intracellular actin filaments of the cell cytoskeleton, which is considered the major factor subjected to cellular morphology and biomechanical response in bone cells. The various types of bone cells do not experience the same ranges of FSS. With respect to osteocytes, which are located within tiny channels, osteoblasts can be found on the surface of soft osteoid and newly formed bone mineral at remodeling sites, i.e. in regions with bigger porosities and, consequently, with reduced fluid flow and fluid shear stress [15, 16]. Reproducing the cell environment inside tissues, in terms of flow rates and distribution, nutrient delivery, waste removal and mechanical stimulation due to fluid shear forces, is a critical point for *in vitro* studies of cells.

Microfluidics, the science of fluid flow at the submillimeter length scale, is an interesting approach to closely mimic this environment. An accurate flow control can be devised with the use of microfluidic devices to test different flow regimes and FSS ranges to which the cell culture is exposed. Moreover, porous scaffolds can be integrated into microfluidic systems in order to obtain 3D flows, which are advisable for the engineering of skeletal tissue construct [13, 17, 18]. The particular combination of biomaterials such as porous scaffolds and microfluidics has the potential to produce more physiologically relevant models for bone regeneration [19, 20].

In our study, we present for the first time a description of the fluidic behavior of the porous scaffold B-HA and the preliminary observations about the modifications induced by different fluid shear stresses applied to osteoblast cells cultured on the B-HA surface. Attention was focused on reproducing natural bone chemistry, morphology and 3D architecture. From the point of view of fluid dynamics, B-HA can be regarded as a network of micro-channels, intrinsically offering the advantages of a microfluidic system: physiological flow conditions can thus be recreated inside the B-HA micro-channels by properly designing the fluid dispensing system. On the other hand, the natural origin of B-HA makes this scaffold peculiar for its geometry, which is neither predictable nor fully customizable. For this reason, there is the need to characterize the behavior of both the B-HA scaffold and the surrounding cells, to understand the mechanisms that control the mineralization process of cultured cells under static and dynamic conditions. For this broad purpose, our experimental workflow comprised three distinct phases: (i) characterization of B-HA scaffold properties, useful to design a fluid perfusion system (bioreactor) and define the fluid shear stresses acting on the cells under dynamic conditions. To this aim, the flow resistance and the morphological features of a number of B-HA samples were analyzed. (ii) Assembling of a simple and inexpensive bioreactor, aimed at exposing the cells cultured in the B-HA scaffold to physiologically relevant flow conditions. (iii) Study of the morphological and biological modifications of osteoblast cells attached to B-HA, as induced by the flow. The effect of two different ranges of shear stress was explored, corresponding to physiological and/or pathological FSS conditions (Low and High FSS) [21–23], and compared to static (i.e. zero-flow) conditions.

## Materials and methods

To study the alterations induced by the flow on osteoblastic cells, B-HA scaffolds were selected as the extracellular matrix for *in vitro* experiments. The application of dynamic flow conditions required, as a first step, the determination of the scaffold fluid dynamic properties in terms of flow resistance. Such a parameter is essential for the integration of the scaffold inside a fluid perfusion system, which was specifically designed to impose different fluid shear stresses. At the same time, for the estimation of FSS, geometrical data are needed about the scaffold structure. Hence, a characterization of the scaffold morphology was also required before cellularization.

### Flow resistance measurements of B-HA scaffolds

In the present study, B-HA scaffolds were adopted in the form of thin cylinders of approximately equal size. The information about phase, chemical composition and mechanical properties of the scaffold is provided in a previous published article [9]. A total number of 12 samples were used under dynamic flow conditions, their diameters ranging from 10.4 to 10.9 mm and heights from 3.7 to 4.8 mm. The complex porous structure of the scaffolds required their flow resistance to be determined experimentally, to obtain accurate values for the subsequent development of the FSS device and for the computation of fluid shear stresses. Preliminary tests were thus performed, based on the measurement of the level variation of a fluid draining through the scaffold.

**Figure 1.**
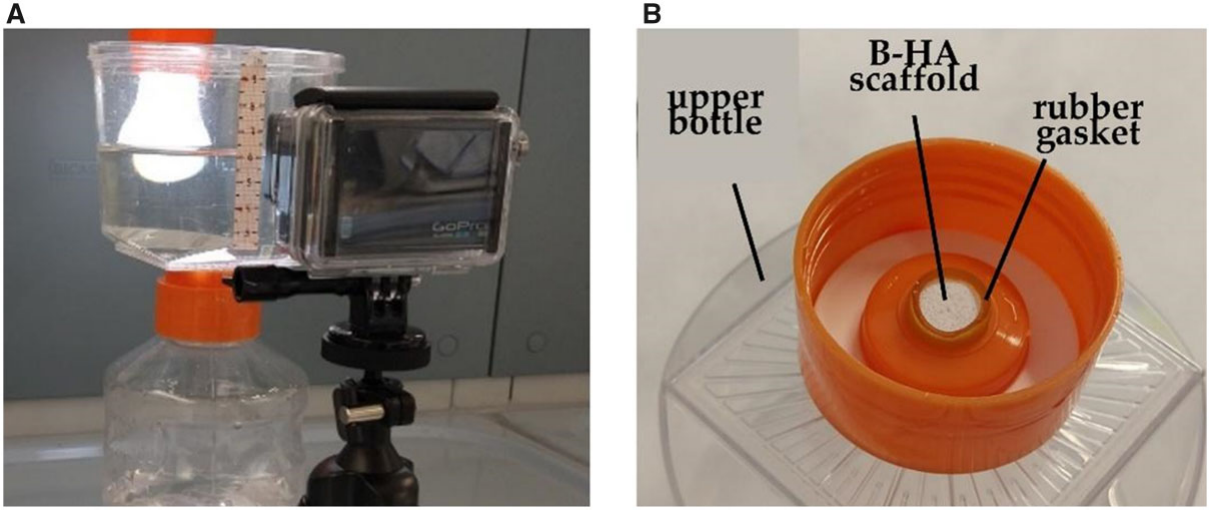
(**A**) Experimental setup for the measurement of scaffold flow resistance at room temperature; (**B**) Scaffold accommodation in the orifice located below the upper bottle.

The experimental apparatus, displayed in Fig. 1A, is based on a commercial vacuum filtration system (Corning 431097, Merck, Darmstadt, Germany) which was slightly modified (i.e. deprived of its membrane filter); in this way, the resulting container provided a simple and low-cost solution to generate flow through the scaffold by means of hydrostatic head. The upper and the lower bottle of the container are connected by a narrow circular passage where the scaffold, equipped with a rubber gasket, fitted into (Fig. 1B). After assembling, the upper bottle was filled with 500 ml of bi-distilled water and the descending liquid level was monitored; a strip of millimeter paper stuck to the container and a simple imaging system, consisting of a camera (GoPro Hero3) and LED light in back lighting, served the purpose. Images were acquired in time-lapse mode, the time interval being set between 2 and 10 s; such a value was adjusted in accordance with the test duration, in order to obtain initial variations of the liquid level of about 1 mm between consecutive frames. Two sets of tests were carried out on each sample at two different ambient conditions, i.e. at room temperature and inside an oven at 37°C, to take into account any possible influence of temperature on scaffold permeability. Tests were repeated 4–5 times per set, re-using the liquid collected in the lower bottle; before a test series, both the container and scaffold were rinsed with bi-distilled water, while, at the end of the series, the liquid was removed and replaced with fresh fluid.

Quantitative analysis of the recorded sequences was performed with the software ImageJ (NIH, USA). Data extraction of the liquid level as a function of time was limited to the conical portion of the upper bottle. The wide-angle lens mounted on the camera caused image distortion, which was compensated by multi-step calibration with reference to the millimeter paper strip. The marginal areas of the frames, suffering the largest distortions, were excluded. In this way, height values were obtained with a maximum error of 2%, mainly ascribable to reading uncertainties associated with the liquid meniscus.

Flow resistance was calculated from the extracted data by applying the Darcy’s law [24], under the realistic assumption of laminar flow:

(1) $$\Delta p\;=\;\frac{L\;\mu }{A\;k}\;Q_{v}\;=\;R_{\mathrm{scaff}}\;Q_{v}$$

In the above equation, *Q*_v_ is the volumetric flow rate, μ is the dynamic viscosity of the water, while *L*, *A*, *k* and *R*_scaff_ are the height, cross-sectional area, permeability and flow resistance of the scaffold, respectively. In the present problem, the pressure drop over the scaffold, Δ*p*, is given by the time varying hydrostatic head. By expressing the fluid volume in terms of the instantaneous liquid height, rearranging, and integrating Eq. (1) between the starting frame and a time *t*, a corresponding value for *R*_scaff_/μ can be derived. See Appendix 1 for the detailed derivation of the scaffold resistance *R*_scaff_. For each sample, the results obtained with this procedure were averaged within the single test duration, and a weighted average was applied to the resulting mean values in order to extract the scaffold flow resistance.

### Morphological characterization of B-HA scaffolds

Following flow resistance tests, the scaffold morphology was examined to retrieve the geometrical information necessary for the quantification of FSS. However, the interconnected porous structure of B-HA is too complex to allow an exact determination of the shear stress values. Hence, for simplicity, the pores were approximated as parallel channels of cylindrical shape, their diameters to be calculated from observations taken on the upper and lower face of the scaffolds. To this aim, images of both the sides of each sample were acquired by a stereomicroscope (Nikon SMZ800, New York, USA, fitting a Plan 1X objective) at the lowest magnification level, and subsequently analyzed with ImageJ. In order to extract information on pore number and size, an automated detection technique already available in ImageJ was applied after image filtration and binarization; the detected pores, measured in terms of cross-sectional area, were then approximated as circles, and their equivalent diameters were calculated. Image resolution was of 7 μm/pixel, and pores with a cross section as small as 47 μm^2^ could be identified with this method.

### Flow shear stress device

**Figure 2.**
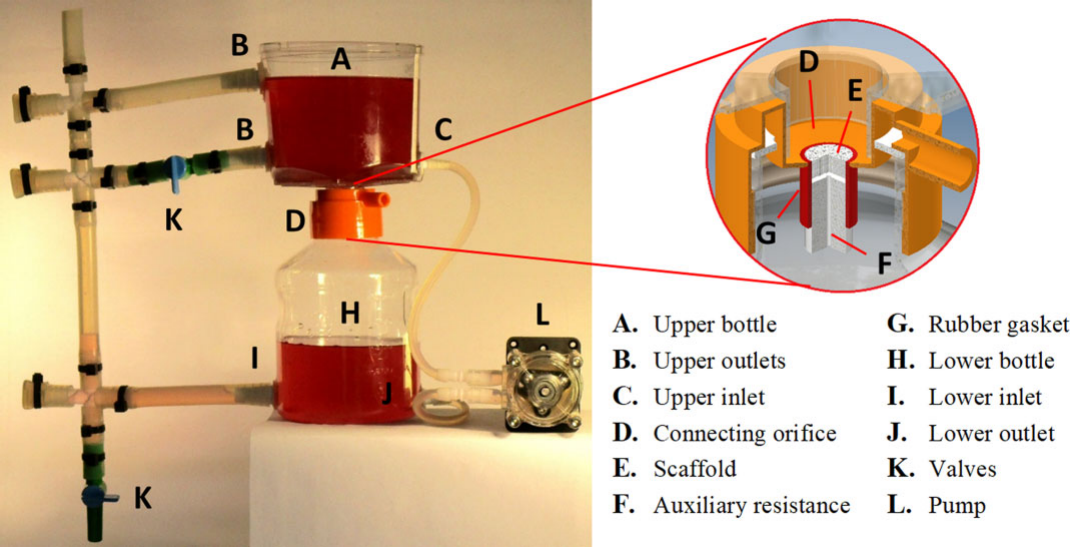
FSS bioreactor used during biological tests.

For the biological tests, a FSS bioreactor was designed and assembled, aimed at generating constant shear stresses over the cells cultured in the B-HA scaffolds. As shown in Fig. 2, the bioreactor was built starting from the same type of container used in the flow resistance tests, with the flow once again driven by hydrostatic head.

In this case, the liquid level was kept constant by an overflow drain and by feeding the liquid column with a peristaltic pump. For this purpose, holes for the fluid inlet and outlet were drilled on both the upper and the lower bottle, which were connected to each other and to the pump via silicon tubing. In particular, the upper bottle was provided with two outlets located at different heights to define two different hydrostatic heads, selectable by manual valves. As in the flow resistance tests, the B-HA scaffold, enclosed in a silicone rubber gasket, was inset in the container orifice. Furthermore, an auxiliary fluidic resistance was placed in series with the scaffold. The auxiliary resistance, a PTFE pad with calibrated holes, was available in three different sizes, with lengths of 16, 9 and 7 mm and internal diameters of 1.0, 0.6 and 0.4 mm, respectively. This allowed for a finer regulation of the flow rate, whose value was calculated from the mechanical energy balance between the liquid free surface and the resistance outlet.

Draining through the scaffold, the liquid flow induces shear stresses on the walls of the pores. By approximating the pore geometry with cylindrical channels of equal length, the following formula can be retained valid:

(2) $$\tau_{i}=\frac{\Delta p_{\text{scaff}}\;D_{i}}{4\;L}$$

where τ_i_ is the individual shear stress in the i-th pore of the scaffold having diameter *D*_i_, Δ*p*_scaff_ is the pressure difference between the scaffold faces, and *L* is the scaffold height. According to Eq. (2), cells cultured inside a scaffold are exposed to different shear stresses, which linearly depend on the pore diameter. If it is assumed that the cell fraction inside a pore is proportional to the pore lateral surface, it is possible to estimate the global shear stress for the whole scaffold as:

(3) $$\tau_{\text{scaff}}=\frac{\sum_{i}\tau_{i}D_{i}}{\sum_{i}D_{i}}$$

that is to say, by doing a weighted average of contributions from individual pores, while taking into account cell repartition.

With reference to this expression, the B-HA samples were divided into two main categories for the tests in the FSS device, defined by two distinct ranges of global shear stress: Low FSS and High FSS, in the order of Pascal and tenths of Pascal, respectively, as shown in Section ‘B-Ha samples: morphology and fluid-dynamical aspects’ [17, 21–23]. This was achieved by means of a specific combination of hydrostatic head and auxiliary resistance, in conjunction with the measured flow resistance and pore distribution of the various scaffolds.

### Cell cultures

Osteoblastic cell line MC3T3 (Subclone 14, Elabscience, Houston, USA) was cultured in a complete medium composed of ALPHA MEM (Corning, New York, USA) supplemented with 10% Fetal Bovine Serum (FBS), 1% L-glutamine and 1% penicillin–streptomycin (100 U ml^−1^–100 µg ml^−1^) (Corning, New York, USA) at 37°C, under a humidified atmosphere of 5% CO_2_. All cells tested negative for mycoplasma and were subsequently grown in fresh medium. The same batch of FBS was used for all the experiments. Cells were cultivated for a minimum of two and a maximum of seven passages in fresh media before experiments were initiated. Cells were counted and seeded at the same density during maintenance and before every experiment. With similar percentage volume of additives, the culture medium can be considered as a Newtonian fluid [25]. As a consequence, the flow resistances of B-HA determined with the method previously described (Section ‘Flow resistance measurements of B-HA scaffolds’) can be retained valid when exposing the cultured scaffold to a flow of the complete medium.

### B-Ha sterilization and preparation

Each sample, placed in ethanol 70% v/v overnight and sterilized by UV radiation prior to use, was placed one per well in a 24-well plate and presoaked in culture medium for 72 h at 37°C. The sample was seeded by carefully dropping 20 µl of MC3T3 cell suspension (5.0 × 10^5^ cells) onto the scaffold upper surface and allowing cell attachment for 20 min in the incubator, before the addition of 1 ml of growing cell culture medium. After 24 h of culture, the seeding procedure was repeated on the scaffold bottom side and the cellularized scaffold was maintained for 48 h in the cell incubator.

### Cell morphological evaluation

For morphological investigation, nine B-HA cellularized scaffolds were tested after 72 h of culture, equally divided in three groups: Ctrl, Low FSS and High FSS. Ctrl represents B-HA scaffolds not subjected to FSS. Low and High FSS samples indicate scaffolds subjected to low and high shear stresses as indicated in ‘Flow shear stress device’ section.

In order to visualize the attached cells, samples were washed with Phosphate Buffer Saline (PBS) 1× for 5 min, fixed with 4% (w/v) paraformaldehyde for 15 min and washed with PBS 1× for 5 min.

#### Fluorescence microscopy

To analyze cells through fluorescence microscopy, before the fixation procedure, cells were stained with Cell tracker Orange CMRA Dye (Thermo Fisher Scientific, Waltham, MA, USA) at a concentration of 5 µM for 30 min. The dye is a rhodol-based fluorophore that should remain primarily in the cytoplasm [26–29]. Where indicated, the cells were stained with CellMask^TM^ Green Actin Tracking Stain (Thermo Fisher Scientific, Waltham, MA, USA) at a concentration of 1 µM for 30 min. The dye can be used to track F-actin in live or fixed cells.

Images were acquired by using an Inverted Confocal Nikon Eclipse A1 fluorescence microscope (Nikon, New York, USA). The projections were scanned at low magnification (×20) to locate the scaffold structure. The increment of the Z-axis optical section was set to 0.5 μm in order to obtain 30–40 continuous images. These were sequentially overlapped along the Z-axis to form a defined stack (one for each representative scaffold). After acquisition, projection of Z-stack images was displayed using the click Z mode. 3D reconstruction and display of cubic imaging were built up by Nikon Elements Software (ver 4.5). Since Deep-Z generates temporally synchronized virtual image stacks through purely digital refocusing, it can be used to match the imaging speed to the limit of the camera framerate, by using the stream mode, which typically enables a short video of several frames per second (see Supplementary section).

#### Field emission gun scanning electron microscopy

The morphology of osteoblasts fixed on the B-HA scaffold was investigated by field emission gun scanning electron microscopy (FEG-SEM) using a Nova NanoSEM™ 450 (FEI Company, Bruker Corporation, Oregon, USA), high vacuum mode, 8 kV and secondary electrons (SEs) detector.

For FEG-SEM analysis, B-HA scaffolds were dehydrated through a graded series of Ethanol (50%, 70%, 80%, 95% and 100% v/v) for 20 min each and left to dry. Finally, B-HA scaffolds were placed on aluminum stubs and coated with a thin layer of gold to improve conductivity.

### Quantitative real-time PCR

Total RNA extraction was performed by the use of the RNeasy kit (Qiagen, Hilden, Germany) according to manufacturer’s instructions. RNA integrity and quantification were analyzed by a spectrophotometric method using a NanoDrop 2000 device (Thermo Fisher Scientific, Waltham, MA, USA). Total RNA (1 μg) was reverse transcribed to cDNA using the QuantiTect Reverse Transcription Kit (Qiagen, Hilden, Germany), according to manufacturer’s instructions. Levels of mRNA were quantitatively determined on a QuantStudioTM 3 Real-Time PCR System (Applied Biosystems, Thermo Fisher Scientific, Waltham, MA, USA) using the QuantiFast SYBR Green PCR Kit according to the manufacturer’s instructions (Qiagen, Hilden, Germany). PCR primer sequences were as follows:

- ALPL forward primer 5′AAGGACATCGCATATCAG-3′,
  ALPL reverse primer 5′-TTCGTATTCCACATCAGTT-3′;
- RUNX2 forward primer 5′-TTAATCCACAAGGACAGA-3′,
  RUNX2 reverse primer 5′-GTAAGACTGGTCATAGGA-3′;
- PTN forward primer 5′- ATGTCGTCCCAGCAATATCAGC-3′,
  PTN reverse primer 5′-CCAAGATGAAAATCAATGCCAGG-3′;
- GAPDH forward primer 5′- CGAGATCCCTCCAAAATCAA-3′,
  GAPDH reverse primer 5′-TTCACACCCATGACGAACAT-3′.

The biological assay was performed on other nine B-HA scaffolds, again divided in three groups as mentioned in ‘Cell morphological evaluation’ section (Ctrl, Low FSS and High FSS), in order to have three technical replicates for each experiment.

Relative gene quantification was performed using the comparative threshold (Ct) method (ΔΔCt), where relative gene expression level equals 2^−ΔΔCt^. The obtained fold changes in gene expression were normalized to the internal control gene GAPDH.

### Statistical analysis

The real-time PCR data were processed by the statistical student *t*-test (one-tailed) and expressed as the means ± standard deviation from three independent experiments. For all tested groups, the statistical significance was set up at *P* values < 0.05.

## Results and discussion

### B-Ha samples: morphology and fluid-dynamical aspects

**Figure 3.**
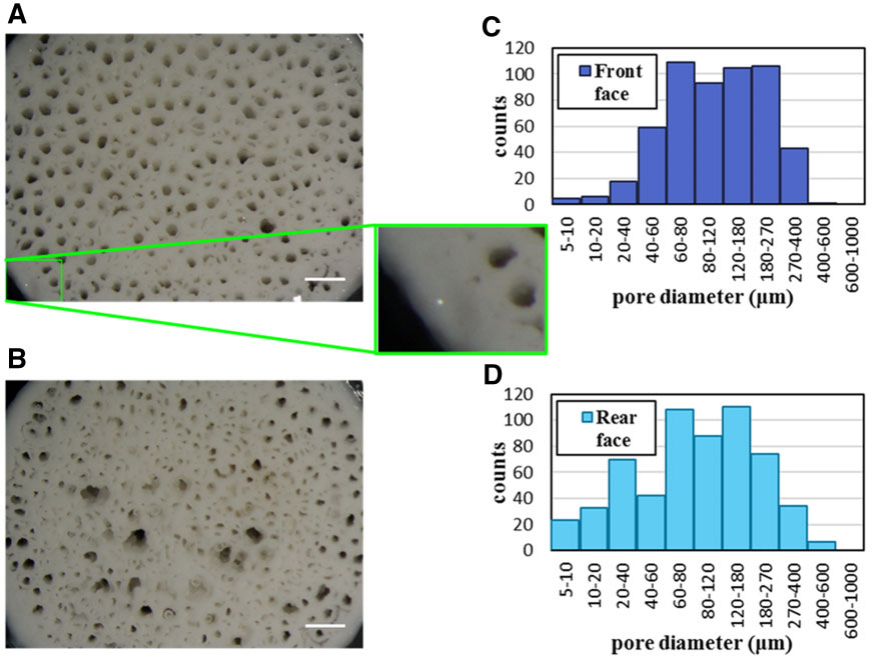
Results of morphological analysis on one of the scaffolds (B-HA 3): images of (**A**) the scaffold front face with an enlargement of a region near the border and (**B**) the scaffold rear face, acquired at the stereomicroscope after the flow resistance tests. Scale bar = 1 mm. Pore diameter distribution on the front **(C)** and rear **(D)** faces, obtained by the images with an accuracy of 7 μm/pixel.

An example of pore distribution, as obtained from the images acquired with the stereomicroscope, is reported in Fig. 3 for one of the scaffold samples. The histogram reveals that more than 500 pores were detected in this case, spanning a wide range of sizes: diameters vary from less than 10 to nearly 600 μm, with almost 70% of the pore population residing in the range 60–270 μm.

The distribution on the front face is similar, although not identical to the one on the rear face: this suggests that approximating the pores with cylindrical channels can be considered valid to some extent, in spite of the greater complexity of the highly interconnected porous geometry typical of these scaffolds. It should be noted that the analyses did not include the outer portion of the scaffold surface, because of the limited field of view of the objective. However, as it can be observed in the blowup in Fig. 3, the scaffold periphery mainly consists of solid structure; hence, the excluded pores would produce only a marginal contribution to the extracted data, especially for what concerns average quantities. The results found on the other samples are qualitatively similar. From the quantitative point of view, differences exist in the amount, maximum size and size distribution of the pores. The total number of the identified pores per sample varies from nearly 400–900, with maximum diameters as low as 250 μm and up to more than 900 μm. Such values for pore dimensions are, on average, smaller than those reported in Ref. [30] for hydroxyapatite scaffolds fabricated with a different method. Scaffold flow resistances, normalized by viscosity, are summarized in Table 1.

**Table 1.** Measured flow resistances of the scaffold samples, normalized by viscosity, and average shear stresses induced in biological tests

	High shear stress group						Low shear stress group					
	B-HA1	B-HA2	B-HA3	B-HA4	B-HA5	B-HA6	B-HA7	B-HA8	B-HA9	B-HA10	B-HA11	B-HA12
R_scaff_/μ (×10^11^ m^−3^)	2.67 ± 0.12	4.1 ± 0.2	3.0 ± 0.2	27 ± 3	6.2 ± 0.6	2.4 ± 0.2	12.06 ± 0.06	4.2 ± 0.3	13.9 ± 1.4	6.7 ± 0.5	6.9 ± 0.3	1.7 ± 0.2
τ_scaff_ (Pa)	2.5	3.1	3.5	2.5	3.2	2.2	0.53	0.55	0.46	0.48	0.43	0.53

Scaffolds were divided in two main groups, according to the investigated range of shear stress. Data are expressed as the mean value ± standard deviation.

The measured values cover more than one order of magnitude, reflecting the differences encountered in pore distribution. Taking advantage of such a wide variety, two main categories of shear stresses could be defined for the scaffolds when tested in the FSS device: a ‘High Shear Stress’ range (High FSS, 2.2–3.5 Pa) and a ‘Low Shear Stress’ range (Low FSS, 0.4–0.6 Pa). As explained in ‘Flow shear stress device’ section, the level of the hydrostatic head and the size of the auxiliary resistance were selected on the basis of the scaffold flow resistance and pore distribution in order to replicate flow conditions as similar as possible within the same category. The resulting global shear stresses, calculated according to Eq. (3), are displayed in Table 1.

### Morphological and biological modification induced by different fluid shear stress

Mechanical stimuli play a vital role in promoting healthy bone development, homeostasis and morphology. Cells can be influenced by different mechanostimuli, which lead to an activation of cellular and inter-cellular responses [17].

Fluid-induced cell stimulation that can lead to an extracellular response can be obtained in two ways: firstly, by direct contact of the cell surface with the moving extracellular fluid, as occurring in the vascular endothelium; secondly, indirectly via the fluid flow through the lacunar network, as experienced by osteocytes subjected to a compressive loading activity. This extracellular cell stimulation leads to an altered cell morphology as well as altered intracellular signal cascades such as changed gene and protein expression pattern. Some previous studies have demonstrated the alteration of osteoblasts (osteocytes precursors), including morphology, expression and release of molecules and cytoskeleton arrangement, after exposure to FSS [31]. However, the results varied due to different frequencies, magnitudes and models (oscillatory or unidirectional) of fluid flow.

Based on this, we wanted to study the morphological and biological effects of FSS on osteoblasts cultured on B-HA microfluidic channels. In this study, MC3T3 cells were cultured on both sides of the B-HA scaffold and after 72 h they were subjected to high (2.2–3.5 Pa) or low (0.4–0.6 Pa) levels of unidirectional FSS for 4 h, using the bioreactor.

**Figure 4.**
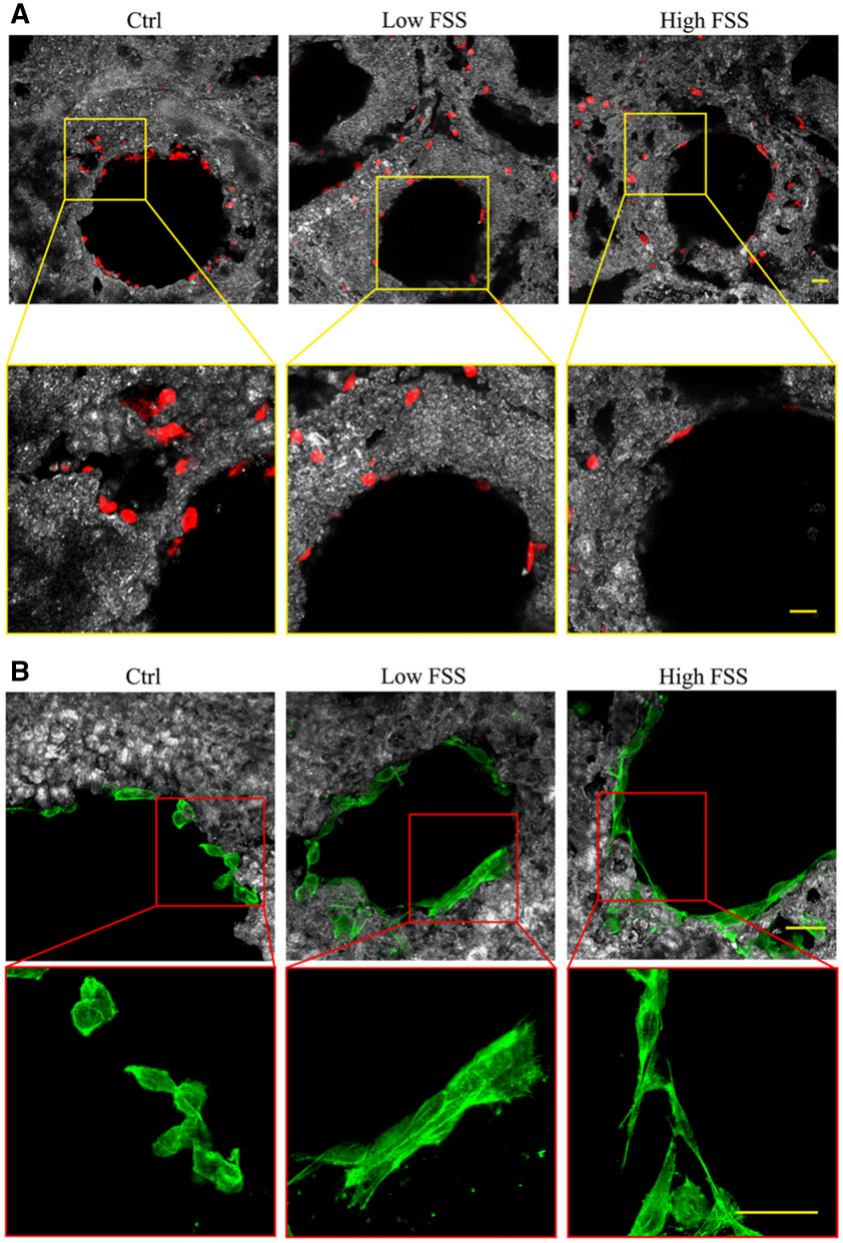
Confocal fluorescence images of three representative cellularized scaffolds divided in three groups (ctrl; Low FSS and High FSS). Osteoblasts were stained with Cell tracker Orange CMRA Dye (**A**, magnification ×20) and CellMaskTM Green Actin Tracking Stain (**B**, magnification ×40) markers. Yellow and red squares show higher magnification of the corresponding images on top. Yellow arrows indicate elongated cells attached to the pore walls. Scale bars: 50 µm.

To assess the alterations taking place inside the cell body, osteoblast cells were next treated with a membrane penetrating fluorescence stain and with a specific stain marker for F-Actin. Because of technical limitations, our confocal microscopy observations were limited to those cells adherent to the pore walls near the top and bottom surfaces of the scaffolds. In Fig. 4, fluorescence microscope images are reported which are representative of the samples exposed to the two levels of FSS (Low FSS and High FSS) as well as to the zero-flow condition (Ctrl). In all the three cases, cells adherent to the B-HA substrate are clearly visible (red and green spots). Moreover, the collected images suggest that osteoblasts tend to elongate in response to both the dynamic flow conditions, with respect to the cells attached to the Ctrl samples. Such a behavior can be better observed in the blow-ups of Fig. 4: a fluorescence signal with a stretched elliptical shape is returned after FSS stimulation by some of the cells localized at the pores edge, (Low and High FSS, panels A and B; in panel A the stretched cells are indicated by yellow arrows), in opposition to the circular signal generally obtained on the reference sample (Ctrl). The modification of the fluorescence signal is symptomatic of cell adaptation to FSS through displacement and deformation of the cytoskeletal filaments (the actin filaments, microtubules and intermediate filaments), which get aligned with the direction of the flow (Fig. 4B) [23].

To further elucidate the details of cells distribution inside the pores, a Z-axis analysis was carried out too with the confocal microscope. Results are illustrated in Supplementary Fig. S1. There, the FSS-triggered effect is demonstrated by the spatial distribution of the fluorescence signal, which varies accordingly to cell orientation. Further spatial information of the cell morphology can be inferred by rotating the image along the X-axis, as provided by the Supplementary Video S2, whereby only cells with an aspect ratio greater than 2:1 were included. The microscopy observations indicate that osteoblasts not only adhere strongly to the scaffold even after the application of shear stresses in the order of the Pascal, but also start out being randomly orientated throughout the intracellular space. Such a response confirms the capability of B-HA substrate to act as a bioactive material, able to promote extracellular matrix interaction through integrins and inorganic components, corroborating the results found in Ref. [8].

**Figure 5.**
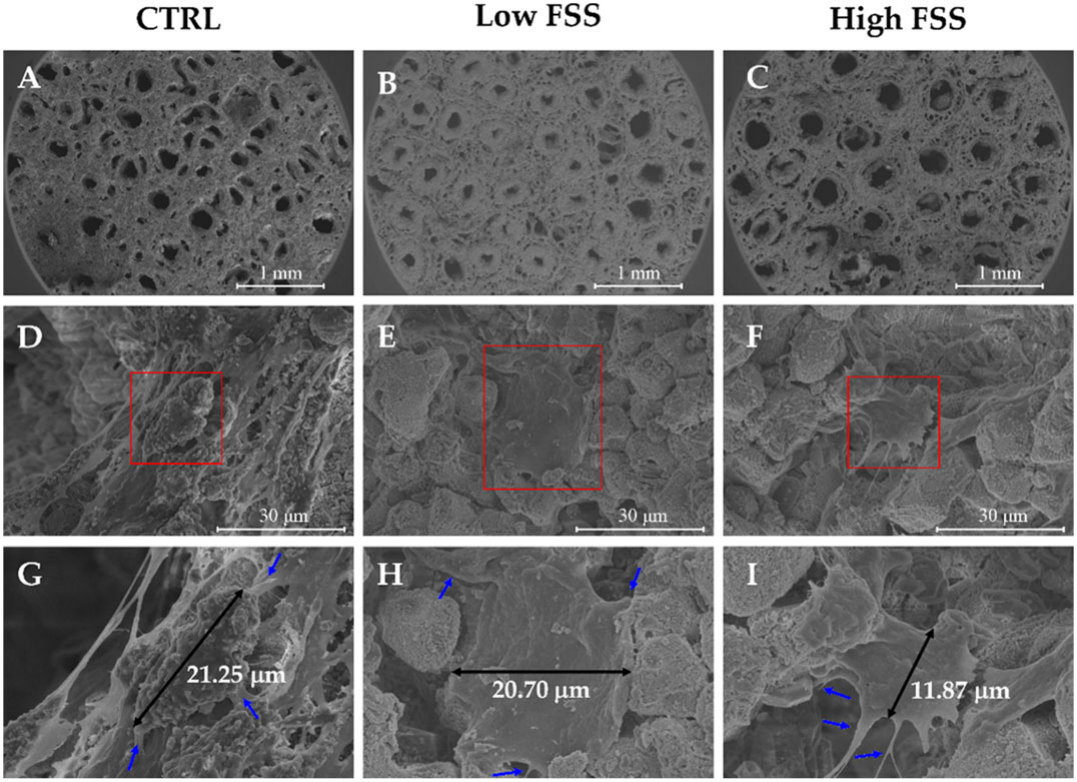
FEG-SEM images of three representative cellularized scaffolds (**A–C**, magnification ×100) divided in three groups (ctrl, Low FSS and High FSS), showing the interconnected porous organization of B-HA scaffolds which faithfully mimics the hierarchical microstructure of the natural bone. The morphological structure of representative cells is illustrated in the absence (**D**) or presence of Low and High FSS (**E** and **F**) (magnification 5 kX). The same cells, visualized in red squares, are shown at higher magnification (10 kX) in panels **G**, **H** and **I**, respectively. Black and blue arrows indicate cell diameters and filopodia, respectively.

The morphological modifications induced by the fluid shear stresses were further explored by means of SEM. The most significant results are reported in Fig. 5. The upper panels (Fig. 5A–C) show low-magnification SEM images of three bare scaffolds, representative of the static (Ctrl) and dynamic (Low and High FSS) treatment. In all these scaffolds, the interconnected porosity is well evident. Images acquired at higher magnification (Fig. 5D–F) provide evidence of the formation, along the scaffold surface, of cytoplasmic extensions from the osteoblasts, which are uniformly and abundantly distributed on the whole area of the scaffold. Their filopodia, indicated by the blue arrows in the enlargements (Fig. 5G–I), are anchored to the surface of the scaffold. The measured cell diameters (black arrows) show that osteoblasts tended to elongate along the direction of the flow. In particular, during high levels of unidirectional FSS treatment, it can be observed that cells underwent a contraction and re-spread process.

These results demonstrated that the growing osteoblasts underwent a process of contraction and re-spreading in response to high FSS (2.2–3.5 Pa) within a period of 4 h. FSS mechanical stimulation alters the interaction between cells and the external environment, which leads to the reorganization of cytoskeleton. As an important mechanosensor, cytoskeleton links to focal adhesion and external cellular matrix and transfers extracellular mechanical stress to intracellular chemical response (outside-in). This early cytoskeleton rearrangement could be interpreted as the prelude to induction of biological events such as changes in gene and protein expression leading to functional change of osteoblasts in response to mechanical stimulation [32]. To determine the response of osteoblasts induced by FSS in terms of proliferation rate, cultured cells were counted using a Burker chamber, after trypsin detachment and trypan blue staining As expected, the data confirmed that the proliferation rate of MC3T3 was not altered by FSS, suggesting that the morphological evidences described above are ascribable only to a cytoskeletal reorganization and not to a self-destruction mechanism of programmed cell death (data not shown).

**Figure 6.**
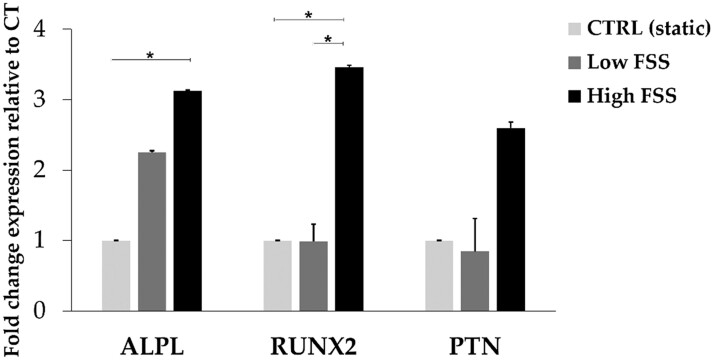
Relative quantification (2^−ΔΔCt^) of ALPL, RUNX2 and PTN mRNA expression as markers of osteogenesis for osteoblasts (MC3T3 cell line). Data are expressed as the mean value ± standard deviation of three samples representative of the static (ctrl) and dynamic (Low and High FSS) flow conditions normalized to the internal control gene GAPDH (*P values ≤ 0.05, *n* = 3).

The influence of the fluid shear stress was also measured by the analysis of the mRNA expression of three differentiation markers, which were selected among the genes mainly expressed at early stage of osteogenesis in MC3T3 cell lines [11, 33–37]. Alkaline phosphatase (ALPL), osteoblast stimulating factor 1 (also known as PTN) and RUNX2 increase upon FSS induction for both the orders of magnitudes tested (Fig. 6), reaching a statistical significant difference in the samples tested with High FSS, as described by several previous studies [11, 32, 38]. This is in line with the ‘early cytoskeletal rearrangement phase’ revealed by the present morphological evaluations. These data corroborate the ability of B-HA bioactive scaffold to promote the differentiation process along osteogenic lineage and the results consolidate the FSS ability as ‘potent driver’ to osteogenic potential.

## Conclusions

Combining osteogenic precursor cells (osteoblasts) and biomaterials based on B-HA, with hierarchical organized structure, is a challenging but promising approach in regenerative medicine, particularly for chronic degenerative disorders affecting bone tissue where the FSS is an important factor that can modify cell fate and produce different proliferation and differentiation patterns. The present study examined the effect of different ranges of FSS on osteoblasts cells cultured inside B-HA scaffolds: Low and High FSS (of the order of tenths of Pascals or Pascals, respectively), corresponding to physiological and/or pathological conditions. Those conditions, closely related to the scaffold morphological features, were obtained by integrating the scaffold in a low-cost bioreactor, which was arranged for the specific problem. Overall, our data revealed that FSS, in conjunction with the biomaterial used as a scaffold, promoted an early stage of cytoskeletal rearrangement, necessary to transduce mechanical forces in cells signaling biological events. Combining growing osteogenic precursors with biomaterial under mechanical high FSS increased RUNX2, ALPL and PTN mRNA expression with respect to those cells induced under Low FSS or static culture conditions. These results confirm that B-HA scaffolds can be effectively coupled to fluidic devices for studying the morphological and biological modifications occurring in osteoblasts when exposed to dynamic flow perfusion conditions, also suggesting that the combination of B-HA with the application of mechanical stimuli via FSS powerfully promote osteogenic induction. This strategy may be considered as a notable improvement for clinical applications to cure musculoskeletal degenerative disorders. As a future development, our research will explore the suitability of combining B-HA scaffolds and microfluidic devices aiming at investigating the response of osteoblasts at different exposure times and levels of fluid shear stress.

## Supplementary data

Supplementary data are available at *REGBIO* online.

## Supplementary Material

rbac034_Supplementary_Data

## Figures and Tables

**Figure A1.**
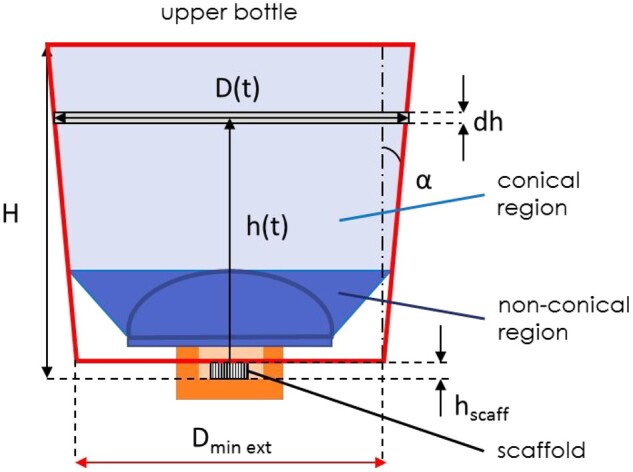
Schematic of the upper container of the filtering device used for the characterization of the scaffold resistance, with indication of the main dimensions. The red line indicates a virtual extension of the conical region down to the upper surface of the scaffold.

## Appendix 1: Derivation of the scaffold resistance

In order to derive the scaffold flow resistance (*R*_scaff_), additional considerations are necessary. With reference to Fig. A1, where a schematic of the upper container of the device of Fig. 1 is reported, along with its main geometrical dimensions, the following relations can be enforced in Eq. (1). The pressure difference is solely given by the hydrostatic head.

Δp=ρ g h(t)

While the volumetric flow rate (*Q*_V_) can be expressed as follows:

$$Q_{V}=\frac{\partial V}{\partial t}=-\pi \frac{D^{2}}{4}\frac{\partial h}{\partial t}=-\pi \frac{{\left(D_{\mathrm{ext}}+2h\mathrm{tantan}\;\alpha \right)}^{2}}{4}\frac{\partial h}{\partial t}$$

The above expression can be considered valid as long as the liquid level lies in the conical portion of the container; *h* is the hydraulic head with respect to the scaffold at the time instant *t*, while *D* is the diameter of the liquid free surface at the instant *t*. The dependence of *D* on *h* can be made explicit by virtually extending the conical region up to the scaffold surface (as highlighted by the red line in Fig. A1).

Substituting the above expressions in the Darcy’s law (Eq. (1)), rearranging and integrating between times 0 and *t* (corresponding to the hydraulic heads *h*_0_ and *h*), respectively, the scaffold flow resistance is obtained:

$$R_{\mathrm{scaff}}=\frac{4\rho \;g\;t}{\pi \left[D_{\mathrm{ext}}^{2}ln\ln\left(\frac{h_{0}}{h}\right)+4D_{\mathrm{ext}}t\mathrm{antan}\;\alpha \left(h_{0}-h\right)+2{\left(\mathrm{tantan}\;\alpha \right)}^{2}\left(h_{0}^{2}-h^{2}\right)\right]}$$
