# Supplementary material for: Liquid flow in scaffold derived from natural source: experimental observations and biological outcome
Source: Regen Biomater. 2022 May 30;9:rbac034. doi: 10.1093/rb/rbac034 (PMC9211004; doi:10.1093/rb/rbac034)
Supplement: rbac034_Supplementary_Data [file rbac034_supplementary_data.zip › Rebuttal 120522.docx]

Dear Editors,

we were pleased to revise our Manuscript ID RB-2022-016 entitled *"Liquid flow in scaffold derived from natural source: experimental observations and biological outcome"* by E. Salerno et al., submitted to Regenerative Biomaterials.

Based on the constructive comments by both the Reviewers, we have reworked specific points and figures in the manuscript in order to fulfill the Reviewers’ inquiries. We have listed below a point-to-point response to Reviewers’ comments.

We hope to have answered exhaustively to all the points raised by the Reviewers. In our opinion, the suggested changes have largely improved the manuscript, and we hope you will now consider it fully suitable for publication in Regenerative Biomaterials.

Answers to the specific Reviewer’s comments are reported below:

**Reviewer: 1**

*R#1: The study of cell proliferation in 3D scaffolds under circulating fluid conditions is undoubtedly interesting topic. The authors have created a unique model that imitates some processes in a living organism. However, I would recommend to read the text again before submitting, as it contains typos and redundant punctuation marks.*

A: We thank the Reviewer for the remark. We have made a general check of the text and corrected typos and punctuation.

*R#1: In addition, it would be great if the authors clarified where they got the B-type hydroxyapatite. If they bought these scaffolds, then it better to provide the characteristics. If they synthesized the scaffolds themselves, then it would be great to provide the xrd analysis, especially since the authors mention the structure of hydroxyapatite.*

A: Thanks for the Reviewer’s assessment. Unfortunately, we cannot mention explicitly the producer of the scaffolds adopted, since they are currently undergoing an industrial development procedure and they are not available on the market yet. Anyway, the mineralogical and chemical characterization of the B-HAs under investigation was performed in the paper “*Tampieri, A. et al. Heterogeneous chemistry in the 3-D state: An original approach to generate bioactive, mechanically competent bone scaffolds. Biomater. Sci. 2019, 7, 307–321, doi:10.1039/c8bm01145a”*, which was already cited in the Introduction of our manuscript (see bibliographic reference n. [9]). We have further emphasized that reference at the beginning of Paragraph 2.1, where we introduce the scaffolds.

*R#1: It is known that hydroxyapatite can be of 2 types: A and B. Why did the authors use the B-type in their work? And is there a difference in cell behavior if A-type is used?*

A: We thank the Reviewer for the observation. The choice of B-type instead of A-type scaffolds was motivated by the method implemented for their production, that is, hydroxyapatite conversion under hydrothermal condition at relatively low temperatures. Such a process allows to obtain only scaffolds where carbonated ions partially replace phosphate ions inside the apatitic structure (B-type). A-type is not achievable in these conditions. We could consider this point in a future work, by changing the process of conversion.

*R#1: Darcy's law is known to be applicable to liquids subject to Newton's law of viscous friction. Did the authors check the fluids used in the work for compliance with this law?*

A: We thank the Reviewer for the remark. The liquid used to determine the flow resistance of the scaffold was bi-distilled water, as specified in Paragraph 2.1. The culture medium here adopted has a Newtonian behaviour too, as confirmed by the literature. Hence, the resistance values extracted with the method proposed in our paper are still applicable when substituting water with complete medium. In order to clarify this point also to the reader, we have explicated such a consideration by adding a sentence in Paragraph 2.4.

*R#1: In Figure 3C, "counts" is used in the histogram axis name, maybe it would be better to recalculate the percentage of pores with one or another diameter? In addition, the histogram bars are superimposed on one another, which is inconvenient to read. I would recommend the authors to redraw.*

A: Thanks for the suggestion. The authors preferred to present the data about the pore diameter distribution as absolute quantities because the total number of the pores is not the same for the two faces of the scaffold (front and rear face). Consequently, providing values in percentage terms would be a little bit misleading. However, as properly recommended by the Reviewer, the bar charts have been separated for the two faces in order to provide a clearer presentation of the data, and Figure 3 has been rearranged.

*R#1: In Figure 4, the stained cells look very blurry. It would be great if the authors added more pictures with a higher magnification, where the cells are seen more clearly.*

A: We thank the Reviewer for the remark. We have modified Figure 4 by taking into account the suggestions of all the Reviewers. Not only the resolution of the images already shown was increased, but we also provided fresh images, acquired after staining the cells with a specific actin target dye which is able to specifically detect cell cytoskeleton. Thanks to this additional methodology, the evidence of the morphological alterations of the cells exposed to FSS was further corroborated.

*R#1: Figure 5 G-I shows SEM photographs of cells, but they look very different (in Figure G, the marked cell looks 3D three-dimensional, while in Figure H - I, the cells are flattened). In addition, MC3T3 cell type is known to be quite large, with an average size ranging from 20 to 50 μm. How can the authors explain that after 4 days of culturing the cells are quite small?*

A: We thank the Reviewer for the observation. With the aim of better clarifying these points and in order to fulfill the Reviewer’s request, the authors performed some additional experiments which have not been reported in the main text but only in this rebuttal letter. Here, the authors also inserted some SEM images to support the discussion about the differences in the cell size and shape.

As highlighted by the Reviewer, it is known from the literature that the size of MC3T3 cells results of nearly 20-50 µm. We would like to point out that this information refers to 2D culture conditions, as for example in the case of cells seeded in a culture plate or glass. In this specific condition, the cells are well attached, flattened and completely adherent to the well plate surface, as visible in the Figure inserted below (Panel A).

On the other hand, when the cells are seeded on a three-dimensional scaffold, they arrange according to the local topography. In our study, MC3T3 cells were seeded on B-HA scaffolds that contain pores of different size and conformation: for the cells adherent to the pore walls, like the one shown in Panel B of the following Figure, the wall proximity alters both the cell shape and extension. For this reason, different cell sizes can be obtained depending on the culturing conditions. The SEM images reported here confirm the values found in the literature (20-50 µm) for the cells adherent to a 2D glass, while for the cells attached to the pores of the B-HA scaffold different dimensions can be reached, as influenced by the pore geometry.

| **(A)** | **(B)** |
| --- | --- |
|  | |
| **Figure:** SEM images of MC3T3 cells which were cultured for 48 hours on a glass surface (A) and on a B-HA scaffold (B) at different magnification shown in the dark table below the figures. The dimensions of the cells are indicated in yellow. Cell protrusions are not included in the measurement. | |

*R#1: In the work, the authors determined the response of osteoblasts induced by FSS, according to the proliferation rate, the cultured cells were counted with trypan blue staining at the experimental points. Since the authors used non-transparent samples in the study and trypan blue is not a fluorescent dye, it is difficult to see the cells. Could the authors explain their technique in more detail.*

A: Thanks to the Reviewer for the request. The authors used the trypan blue staining after the trypsin detachment. The count was made at the end of the experiments using a Burker chamber. A suitable explanation has been added in Paragraph 3.2.

**Reviewer: 2**

*R#2: The manuscript “Liquid flow in scaffold derived from natural source: experimental observations and biological outcome” submitted to the journal RB exhibited the study on the fluid-induced cellular changes, and therefore to present a preliminary study on the effect of fluid on bone cells. It should be a meaningful research work, but the content of this manuscript is relatively simplified. I would like to recommend acceptance of this manuscript only after major revision. 1. Section 2.6, it would be not scientific enough to examine cell morphologies when the cells reach 100% confluence. High growth density will certainly affect the morphology and function of cells.*

A: We thank the Reviewer for theremark. Actually, we have noticed that the statement about cell confluence was a little bit misleading. In fact, as known from the literature, the time indicated for MC3T3 cell cultures to reach 100% confluence (72 hours) refers to 2D culture conditions, where cells are free to colonize the entire plate surface. In a 3D scaffold like the B-HA studied in this paper, the cells distribute not only on the front and the rear surfaces, but also within the pores. Therefore, due to the larger contact area available, the cells cultured on B-HA will not reach 100% confluence within the 72 hours of culturing time. Hence, in order to avoid confusion, the sentence in the paper has been modified by simply deleting the reference to the confluence time, as it does not provide essential information.

*R#2: How is the real shear stress of the bone and/or bone cells? Is there any literature that can provide efficient data to support the two main categories of shear stresses that investigated in this manuscript?*

A: We thank the Reviewer for the observation. References to some papers supporting the choice of the range of Shear Stress applied in this study were already provided at the end of Paragraph 2.3. In particular, the cited articles are the following:

17- Wittkowske, C.; Reilly, G.C.; Lacroix, D.; Perrault, C.M. In Vitro Bone Cell Models: Impact of Fluid Shear Stress on Bone Formation. *Front. Bioeng. Biotechnol.* **2016**, *4*, doi:10.3389/fbioe.2016.00087.

21- Coughlin, T.R.; Niebur, G.L. Fluid shear stress in trabecular bone marrow due to low-magnitude high-frequency vibration. *J. Biomech.* **2012**, *45*, 2222–2229, doi: 10.1016/j.jbiomech.2012.06.020.

22- Ban, Y.; Wu, Y. ying; Yu, T.; Geng, N.; Wang, Y. yue; Liu, X. guang; Gong, P. Response of osteoblasts to low fluid shear stress is time dependent. *Tissue Cell* **2011**, *43*, 311–317, doi: 10.1016/j.tice.2011.06.003.

23- Riehl, B.D.; Lee, J.S.; Ha, L.; Kwon, I.K.; Lim, J.Y. Flowtaxis of osteoblast migration under fluid shear and the effect of RhoA kinase silencing. *PLoS One* **2017**, *12*, 1–16, doi: 10.1371/journal.pone.0171857.

*R#2: Authors should provide clear data presentation in Table 1 to distinguish the two groups.*

A: Thanks for the observation. The table was modified accordingly.

*R#2: As for the fluid influenced cell morphology changes, it is hard to tell obvious difference between the control group and the other two groups. Authors should provide much more clear or magnified clsm images. Or, cytoskeleton instead of the membrane should be stained to present “elongated cells”.*

A: Thanks a lot to the Reviewer for the constructive suggestion. The Figure 4 has been modified by taking into account the indications of all the Reviewers. Not only the resolution of the images already shown was increased, but the authors also provided fresh images acquired after staining the cells with a specific actin target dye which is able to specifically detect cell cytoskeleton. Thanks to this additional methodology the morphological alterations of the cells exposed to FSS were further enhanced.

To elucidate the effects of this second marker on the cells, the authors performed some ancillary experiments on MC3T3 cells cultured on a 2D glass surface for 24 hours. Some of the obtained images are presented in the Figure reported below, which shows how different cell components are targeted by the two stains used in our study. In Panels A and B, cells marked with the red stain (Cell tracker, Orange CMRA Dye) are visible, but no specific structural component is detectable even at higher magnification. Panels C and D are relative to cells marked with CellMask Green Actin Tracking Stain. The actin cytoskeleton is clearly visible in this case, and the cells appear flattened and with a regular microfilament meshwork.

**D**

**C**

B

**A**

**Figure:** Fluorescence microscopy of MC3T3 cells which were stained for 30 min with different cell trackers and cultured for 24 hours on a glass surface. The images shown were obtained at magnification 20X (A-C) and 40X (B-D).

*R#2: Besides 4 hours of exposure to the fluid, any other periods have been investigated? Longer incubation time may lead to different results because the cells need time to adapt the environmental changes.*

A: Thanks to the Reviewer for the consideration about longer time of flux exposure. At the beginning of our project, the experiment was set up at 4 hours to avoid cell detachment. In the future, the authors are planning different fluxing time.

*R#2: Format of the references should have been well uniformed.*

A: Thank to Reviewer for the remark. We have checked and corrected the format of the references. The authors remark that there are some references that have different format.

**#8:**

Sprio, S.; Sandri, M.; Iafisco, M.; Ruffini, A.; Panseri, S.; Montesi, M.; Adamiano, A.; Dapporto, M.; Tampieri, A. *Developing biocomposites as scaffolds in regenerative medicine*; Biomedical Composites (Second Edition); 2017, 543-568. ISBN 9780081007525

**#10:**

Tampieri, A.; Sprio, S.; Ruffini, A. Large 3D porous scaffolds made of active hydroxyapatite obtained by biomorphic transformation of natural structures and processes for obtaining them. Patent; WO/2017/021894; 20*17*.

**#24:**

Straughan, B. *Convection in porous media*; 2008; Vol. 165; ISBN 9780387290966.
